# Infrared Optical Functions of Water Retrieved Using Attenuated Total Reflection Spectroscopy

**DOI:** 10.1177/00037028221128813

**Published:** 2022-10-05

**Authors:** Luis G. Vieira

**Affiliations:** 1 Centro de Física das Universidades do Minho e do Porto (CF-UM-UP), Laboratório de Física para Materiais e Tecnologias Emergentes (LaPMET) and Departamento de Física, 56059 Universidade do Minho, Braga, Portugal

**Keywords:** Infrared spectroscopy, attenuated total reflection, ATR, spectrum modelling, optical functions, water

## Abstract

Comprehensive modeling of non-polarized infrared attenuated total reflection spectrum based on Fresnel equations and wavenumber-dependent dielectric function models of isotropic materials is shown to be a suitable and easy methodology to retrieve optical functions. The scheme is completely general and can be used even for strong dispersion and absorption resonances. Attenuated total reflection spectra in liquid water, measured in the spectral region 100–4400 cm^−1^ with diamond and germanium as internal reflection elements, were used to illustrate and evaluate the method. The refractive index of water computed from the dispersion analysis is critically compared with literature data.

## Graphical abstract

**Graphical Abstract:**
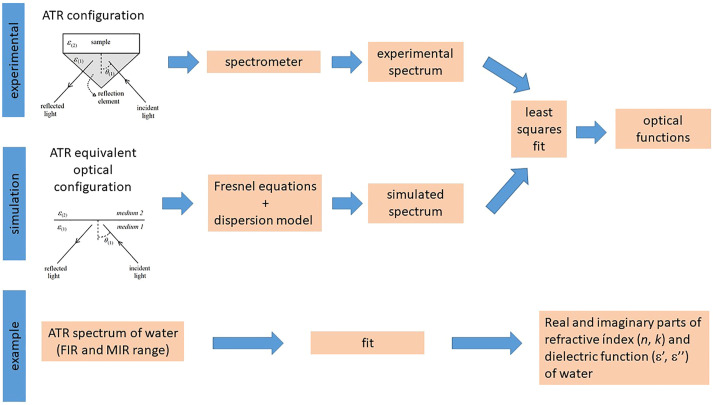

## Introduction

As all the information needed for a quantitative description of the optical properties of a certain material resides in its frequency-dependent optical functions (complex refractive index or dielectric function), efforts to access these parameters are central in spectroscopic investigations. The transmittance technique has been used profusely to perform this task but raises practical difficulties in numerous situations concerning the preparation of the samples. Now, attenuated total reflection (ATR) spectroscopy, also known as internal reflection spectroscopy,^1,2^ has become a substitute for transmission in many circumstances. It presents several advantages over transmission spectroscopy, above all, because of the assembling easiness. An historical overview of the technique can be found in Mirabella.^3^

Attenuated total reflection spectroscopy is eminently a reflectance spectroscopy, as pointed out since its advent.^1,4^ Therefore, the analogy with transmission measurements must be done with caution, since spectra can be substantially affected by changes in the angle of incidence^1^ or of the internal reflection elements.^1,5^ Moreover, a strong dispersion–absorption can cause peculiar effects in the ATR spectrum.^5,6^

Many users of ATR spectroscopy tend to look, incorrectly, at the technique as a modified version of transmission spectroscopy. Furthermore, very often it is assumed that low absorption conditions are satisfied even without confirmation and the variation of the refractive index throughout the width of the absorption bands is unjustifiably neglected.

It is indeed correct that ATR spectra are very similar to transmission spectra under these conditions,^1^ but the technique is not limited to these situations and no approximations are needed as long as the spectra are modeled as of reflectance type with appropriate dielectric function models. The approach should be analogous to standard spectral modeling using Fresnel reflection coefficients and dielectric function models widely used to retrieve the optical functions from external reflectance spectra. The fitting technique has been discussed extensively and goes back to the emergence of systematic infrared characterization of bulk crystals using the classical oscillator dispersion formula^7–9^ and later using other dielectric functions.^10–12^

In what concerns ATR spectroscopy, a comprehensive fitting of the spectra using this same approach to extract the optical functions is seldom used. Examples of such an approach are the works of MacDonald and Bureau^13^ and also Milosevic et al.^14^ The works of Piro et al.,^15^ Guida et al.,^16^ Balan et al.,^17^ and Aufort et al.^18^ partially follow that path by using dielectric dispersion models to simulate the ATR spectra, but the model parameters were computed from other spectroscopic techniques.

The present work was undertaken with three aims. First, to stress that proficient interpretations of ATR spectra are best performed by extracting the optical functions from standard reflectance simulations, second, to point out the ease of such a procedure, and third, to evaluate the method in an extended infrared region using ATR measurements of water taken with different internal reflection elements. The infrared optical properties of water are well known, and many infrared spectroscopic studies have been performed over the last decades. An exemplary case of exhaustive determination of the complex refractive index was performed by Downing and Williams^19^ based on transmission and standard specular reflectance at near normal incidence. In the present work, it is shown that optical functions of similar quality can be obtained from the much less laborious technique of ATR.

## Measurement Configuration and Spectrum Modeling

**Figure 1.**
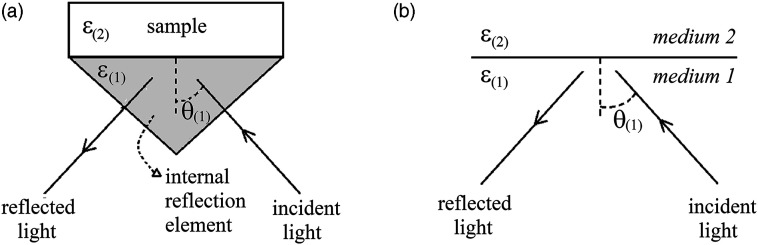
(a) Configuration of the single reflection attenuated total reflection system: The sample of dielectric constant ε_(2)_ is brought into contact with an internal reflection element of dielectric constant ε_(1)_. (b) Equivalent optical arrangement of the attenuated total reflection configuration system.

Typical single reflection ATR setups consist of transparent, high-refractive index, and non-dispersive material, that is, the internal reflection element (IRE) that is traversed by the spectrometer beam before and after reflection at the interface with a second medium, as illustrated in Fig. 1a. Notice that this configuration does not guarantee that internal reflection takes place in all situations: if the sample exhibits strong absorption bands, the increased refractive index might supplant that of the IRE in certain spectral regions (in this case the reflection is external). The measurement procedure to obtain the ATR spectrum consists in dividing the reflected signal when the sample is in contact with the IRE and the reflected signal acquired with no sample. Apart from the reflection at the interface with the sample, all the optical effects occurring inside the spectrometer, including the reflections at the entrance and exit of the IRE, are canceled out when rationing to the background signal. Thus, the reflectivity in this ATR configuration (resulting from either internal or external reflection) can be modeled by considering the equivalent optical system shown in Fig. 1b). This equivalent arrangement consists of two media, with parallel interfaces: a semi-infinite medium 1 (material that constitutes the IRE) and a semi-infinite medium 2 (sample).

Only the situation where media 1 and 2 are isotropic and homogenous will be considered. Let ε_(1)_ and ε_(2)_ designate the complex dielectric functions of these media, respectively. The complex refractive index of medium *j* is

(1) $${\tilde{n}}_{\left(j\right)}=n_{\left(j\right)}+ik_{\left(j\right)}$$

and is related to the complex dielectric function

(2) $$\varepsilon_{\left(j\right)}=\varepsilon_{\left(j\right)}^{\prime }+i\varepsilon_{\left(j\right)}^{\prime \prime }$$

by

(3) $${\tilde{n}}_{\left(j\right)}=\sqrt{\varepsilon_{\left(j\right)}}$$

Let θ_(1)_ and θ_(2)_ denote the angles of incidence and refraction, respectively. The complex reflection coefficients of this system, for *s* and *p* polarized light, *r*_*s*_ and *r*_*p*_, respectively, can be calculated from the boundary conditions for electric and magnetic fields and are given by the Fresnel equations (e.g., Born and Wolf^20^):

(4) $$r_{s}=\frac{\sqrt{\varepsilon_{\left(1\right)}}\cos\theta_{\left(1\right)}-\sqrt{\varepsilon_{\left(2\right)}}\cos\theta_{\left(2\right)}}{\sqrt{\varepsilon_{\left(1\right)}}\cos\theta_{\left(1\right)}+\sqrt{\varepsilon_{\left(2\right)}}\cos\theta_{\left(2\right)}}$$

(5) $$r_{p}=\frac{\sqrt{\varepsilon_{\left(1\right)}}\cos\theta_{\left(2\right)}-\sqrt{\varepsilon_{\left(2\right)}}\cos\theta_{\left(1\right)}}{\sqrt{\varepsilon_{\left(1\right)}}\cos\theta_{\left(2\right)}+\sqrt{\varepsilon_{\left(2\right)}}\cos\theta_{\left(1\right)}}$$

To express these equations only in terms of θ_(1)_ one can use the relation between this angle and θ_(2)_ (Snell’s law):

(6) $$\sqrt{\varepsilon_{\left(1\right)}}\sin\theta_{\left(1\right)}=\sqrt{\varepsilon_{\left(2\right)}}\sin\theta_{\left(2\right)}$$

conjugated with the trigonometric identity sin^2^*x* + cos^2^*x* = 1. The formulas written above are valid for the general case of complex angles and complex dielectric functions (absorbing media), but in the present situation medium 1 is transparent so that ε_(1)_ is real.

The reflectance for *s* and *p* polarizations are given by

(7) $$R_{s}={\left|r_{s}\right|}^{2}$$

and

(8) $$R_{p}={\left|r_{p}\right|}^{2}$$

respectively.

Since many measurements in ATR configuration are performed with non-polarized light, it is important to treat the case of non-polarized reflectance, *R*. For completely unpolarized light (natural light), this situation is contemplated by considering 50% *s*–50% *p* polarization of the incident beam:

(9) $$R=\frac{1}{2}\left(R_{s}+R_{p}\right)$$

However, in a typical Fourier transform infrared (FT-IR) spectrometer, the infrared beam is partially polarized, as is the case of the present work. In these circumstances, Eq. 9 is an approximation.

It is a common procedure to convert ATR spectra using the same absorbance transform used for transmission spectra. In such a case the target function to be modeled is the absorbance transform of ATR:

(10) $$A_{T}=-\log\left(R\right)$$

where “log” represents the base 10 logarithm. Of course, this is not a true absorbance, as noticed by Milosevic.^2^

The reflectance spectrum or the absorbance transformed spectrum can be modeled by using Eqs. 4 to 10 and an appropriate model for the dielectric function of the two media. Various models may be considered, as long as the causality principle is not violated: the real and imaginary parts of the dielectric function must be related by the Kramers–Kronig relations (e.g., Lucarini et al.).^21^ Since the IRE (medium 1) is chosen to be transparent and without dispersion, the corresponding dielectric function is an a priori known real constant. From now on the real and imaginary parts of the dielectric function of the sample (medium 2) will be denoted simply by ε^′^ and ε^′′^, respectively. Similarly, the real and imaginary parts of the refractive index will be represented merely by *n* and *k*.

In the examples that come next the complex dielectric function of medium 2 (sample) is taken to depend on wavenumber ($\tilde{\nu }$) according to the factorized form of the dielectric function:^10^

(11) $$\varepsilon \left(\tilde{\nu }\right)=\varepsilon_{\infty }\overset{n}{\underset{j=1}{\prod }}\frac{\Omega_{Z_{j}}^{2}-{\tilde{\nu }}^{2}+i\tilde{\nu }\gamma_{Z_{j}}}{\Omega_{P_{j}}^{2}-{\tilde{\nu }}^{2}+i\tilde{\nu }\gamma_{P_{j}}}$$

where Ω_*Zj*_ and Ω_*Pj*_ are the zeroes and poles of the dielectric function, respectively, γ_*Zj*_ and γ_*Zj*_ are damping coefficients, and ε_∞_ is the high-wavenumber dielectric constant (usually at optical frequencies). This is a very versatile dielectric function with the capability of describing diverse dielectric responses. It is often used to describe the optical properties of crystals to extract the parameters of the optical modes (in that case Ω_*Pj*_ and Ω_*Zj*_ are identified with the frequencies of the transversal and longitudinal optical modes, respectively). However, if it is used in situations where the dielectric response is more complex, like in amorphous materials, the physical meaning of the mode parameters may have to be dropped.

**Figure 2.**
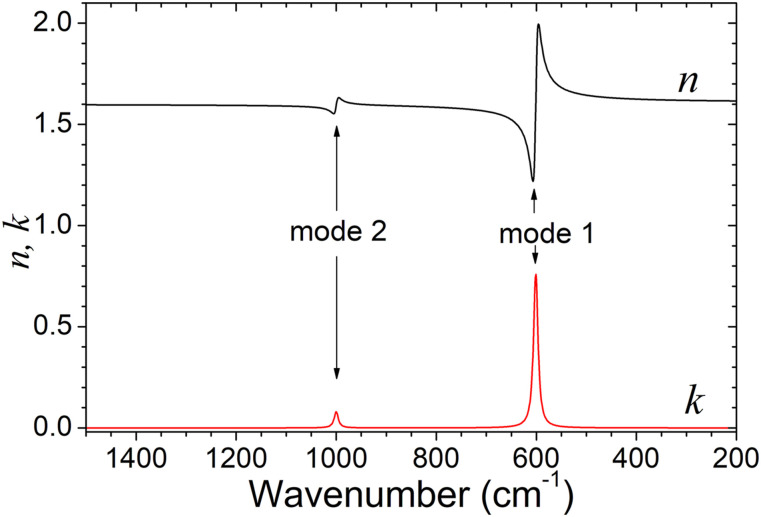
Real (*n*) and imaginary (*k*) parts of the refractive index of a model medium with two well-separated modes. The parameters associated with the dielectric function (Eq. 11) are the following: Ω_*P*(1)_ = 600.0 cm^−1^, Ω_*P*(2)_ = 1000.0 cm^−1^, Ω_*Z*(1)_ = 605.0 cm^−1^, Ω_*Z*(2)_ = 1000.5 cm^−1^, γ_*P*(1)_ = γ_*Z*(1)_ = γ_*P*(2)_ = γ_*Z*(2)_ = 10.0 cm^−1^, and ε_∞_ = 2.56.

From the point of view of spectrum modeling, there is no fundamental difference between different types of reflection spectra: external, internal, or attenuated total reflection. Differences arise only from the relative magnitude of the refractive indices of the two involved media. To illustrate this, consider a model medium as the sample medium (medium 2) placed at the interface with IRE (medium 1) of different refractive indices. The model medium is described by a dielectric function of the Eq. 11 type with two well-separated modes at wavenumber Ω_*P*(1)_ = 600.0 cm^−1^ and Ω_*P*(2)_ = 1000.0 cm^−1^, exhibiting very different dielectric strengths. The remaining model parameters are Ω_*Z*(1)_ = 605.0 cm^−1^, Ω_*Z*(2)_ = 1000.5 cm^−1^, γ_*P*(1)_ = γ_*Z*(1)_= γ_*P*(2)_ = γ_*Z*(2)_ = 10.0 cm^−1^, and ε_∞_ = 2.56. Figure 2 shows the corresponding real and imaginary parts of the refractive index as a function of wavenumber. The refractive index at high wavenumber is ε_∞_^1/2^ = 1.60.

**Figure 3.**
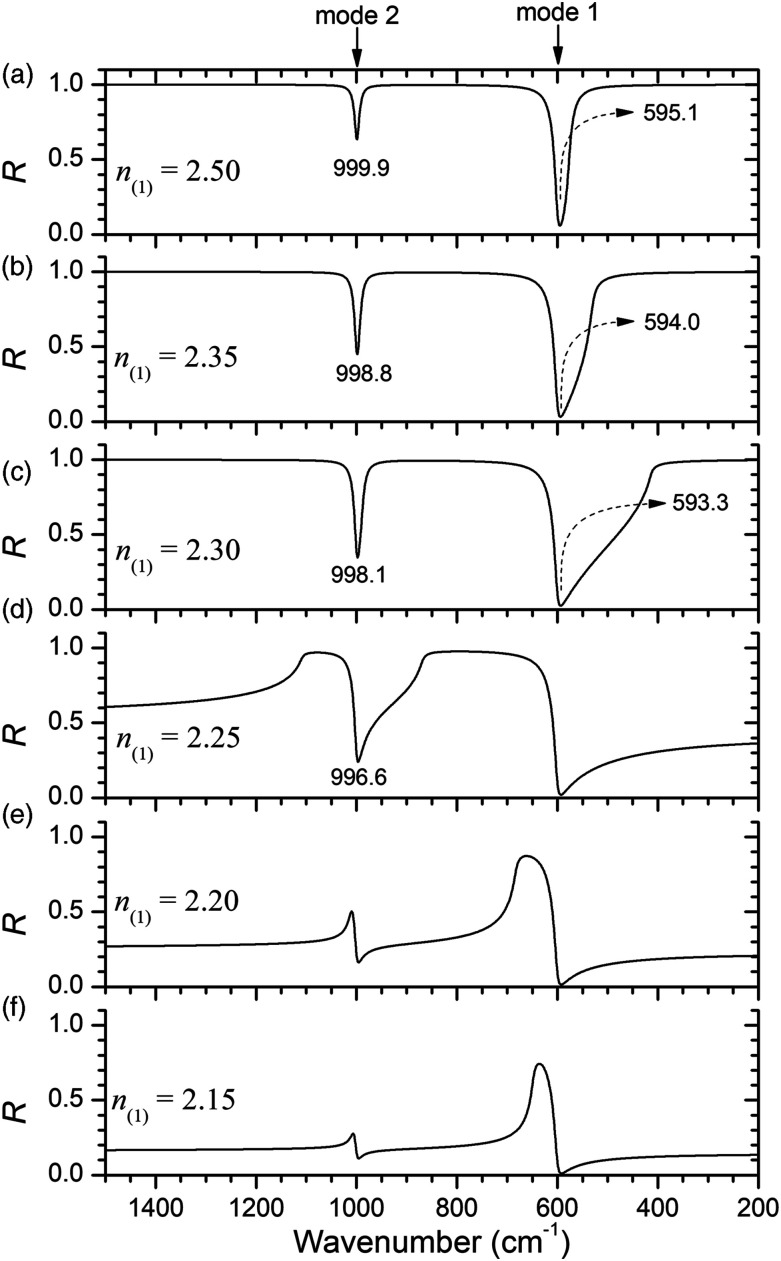
Variation of the reflectivity (*R*) spectra of model medium (the same as in Figure 2) with the index of refraction of the internal reflection element (*n*_(1)_).

Figure 3 illustrates the effect of the variation of the refractive index of the IRE on the non-polarized reflectivity at the interface with the sample medium. The spectra are simulated by using Eqs. 4 to 9 for an incidence angle of 45°. Examining the Fresnel equations (Eqs. 4 and 5), it can be seen that there is a simple relation between the *s-* and *p*-polarized reflectance (*R*_*p*_ = *R*_*s*_^2^) for this particular angle of incidence (45°), regardless of the refractive indices of the two media.^2^ Hence, the major contribution to the unpolarized reflectance comes from the *p*-polarized spectrum. Total internal reflection occurs in the high wavenumber transparent region if *n*_(1)_sin(45°) ≥ 1.60, that is, if *n*_(1)_ ≥ 1.265. This effect is observed in spectra in Figs. 3a–3c. On the contrary, the spectra of Figs. 3d–3f, with profiles quite different from the typical ATR spectra, do not exhibit total reflection at high wavenumber. The sequence of graphs from Figs. 3a–3f illustrates how the spectrum evolves from external reflection to attenuated total reflection.

The spectra in Figs. 3a–3c evince that the shape and position of the ATR dip depend very much on the relative refractive indices of media 1 and 2. Only when the difference between those refractive indices is high the bands are symmetric and located at the wavenumber of the mode. For instance, in spectrum (c) the band of the stronger mode (mode 1) is asymmetric and its minimum shifted (about 7 cm^−1^) to a lower wavenumber with respect to the true 600 cm^−1^ mode wavenumber. As the index of refraction of the IRE increases (spectra in Figs. 3a and 3b), the band becomes more symmetric, and its location approaches the expected wavenumber. The same behavior is observed for the weaker mode 2 but in less extent. The ATR band of mode 2 is already approximately located at the right wavenumber of the mode (1000.0 cm^−1^) in spectrum (a). These calculated spectra exemplify quite well how different are ATR and transmission spectra, despite their resemblance. So, in general, it is not correct to analyze the ATR bands like absorption bands. Unless a simulation of the spectrum is performed, a band like the one in spectrum (c) corresponding to mode 1 could be confused with a superposition of bands and its wavenumber wrongly estimated. Even in spectrum (a), where that same band is symmetric, a shift of about 5 cm^−1^ in respect to the true wavenumber of the mode is still observed.

## Determination of the Optical Functions of Liquid Water

### Preliminary Remarks

Liquid water is quite well suited to illustrating the relevance and testing the reliability of the method of modeling the entire ATR spectra to retrieve the dielectric function. First, its infrared optical properties are very well known, making it easy to assess the accuracy of the method. Second, the infrared spectrum of water is simple to model: liquid water is an isotropic material and has only a few infrared absorption bands, although some are extremely intense and complex to interpret (resulting from overlapping of modes). Third, the liquid state of water ensures that good optical contact with the IRE is achieved, allowing us to consider the equivalent optical configuration represented by only two media (as described in the Measurement Configuration and Spectrum Modeling section above) and to discard any spurious effects caused by an eventual poor optical contact.

### Experimental

Measurements were carried out with distilled water used for standard laboratory applications. The ATR spectra were taken at a nominal angle of incidence of 45° with a Quest ATR Accessory (Specac) mounted in an FT-IR spectrometer (Bruker IFS 66V). For comparison, the results from two ATR crystals (used as IRE) with significantly different refractive indices were employed: an extended range diamond crystal and a germanium (Ge) crystal, with transmission ranges (provided by the manufacturer) 40–10** **000 cm^−1^ and 480–5500 cm^−1^, respectively. However, it was found that in the specific conditions of measurement poor quality spectra were obtained below 100 cm^−1^ for diamond and 600 cm^−1^ for Ge. It is assumed that both crystals are transparent (ε^′′^_(1)_ ≈ 0) and present a nearly constant refractive index (2.40 and 4.00 for diamond and Ge, respectively) in the scanned spectral regions (100–4400 cm^−1^ and 600–4400 cm^−1^ for diamond and germanium, respectively). These are the refractive indices at 1000 cm^−1^ provided by the manufacturer. The reasonableness of the approximation of complete transparency and no dispersion of the refractive index of the IRE along the whole studied spectral ranges is discussed in the next sub-section.

All measurements were performed at room temperature in vacuum using a Globar source. Different beam splitters and detectors were employed for distinct wavenumber ranges. A KBr beamsplitter and a deuterated triglycine sulfate (DTGS) detector with a KBr window were used to cover the mid-infrared (MIR) region (500–4400 cm^−1^). The lowest wavenumber region (100–500 cm^−1^) was examined with a 6μM8 Mylar beamsplitter and a DTGS detector with a polyethylene window. As typically observed in FT-IR spectrometers, the beam entering into the ATR accessory was partially polarized, hence composed of unpolarized and polarized light. The average value of the wavenumber-dependent fraction of polarized light (degree of polarization) was estimated to be about 17%. This calculation was based on the maximum and minimum intensities of transmitted light through a polarizer when it rotates (e.g., Chipman et al.).^22^ The nominal resolution for the 100–500 cm^−1^ and 500–4400 cm^−1^ spectral ranges was 2 and 4 cm^−1^, respectively.

The background spectral intensity was measured before the sample placement when light impinges from the ATR prism to vacuum satisfying the conditions of total internal reflection. The attenuated spectral intensity was recorded when the sample was brought into contact with the prism. The reflectivity was calculated as the ratio of the later and the former spectral intensities.

Finally, the following corrections to the spectra were made: since at the highest wavenumber region, in the transparent region, total reflection should be observed, the MIR spectra acquired with diamond and Ge ATR crystals were multiplied by 1.0184 and 1.0101, respectively, to bring the reflectivity close to one at high wavenumber; the spectrum recorded with diamond IRE in the region 100–500 cm^−1^ was multiplied by 0.9283 so that this spectrum matches with the MIR spectrum in the intersection region between 400 cm^−1^ and 500 cm^−1^.

### Results and Analysis

**Figure 4.**
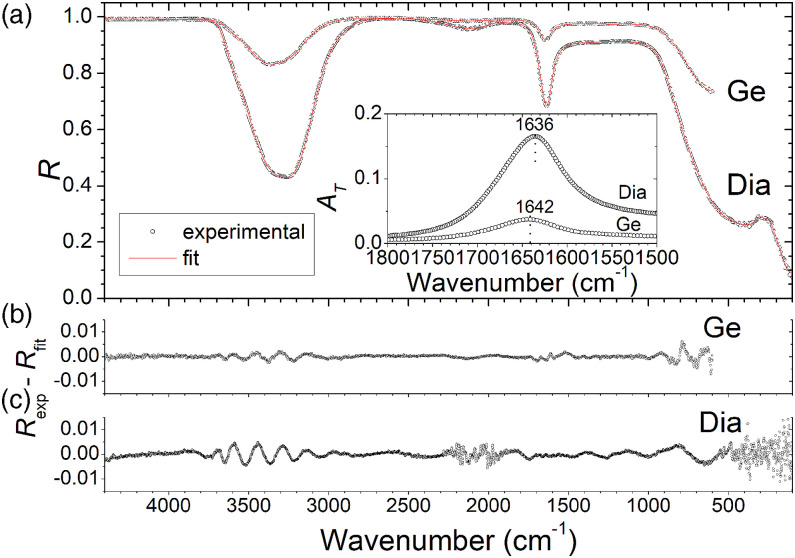
(a) Experimental (dots) and fitted (lines) attenuated total reflection spectra of water acquired with germanium (Ge) and diamond (Dia) as internal reflection elements. The inset shows the absorbance transform (calculated according to Eq. 10) of the experimental spectra in the region around 1640 cm^−1^. The fitting parameters are shown in Tables A1 and A2 of the Appendix. Panels (b) and (c) show the residuals, that is, deviations of experimental and calculated curves (*R*_exp_ – *R*_fit_). For the experimental data, fitted spectra, and optical functions of water, see the research data files in the Supplemental Material.

The measured ATR spectra of water acquired with diamond, covering the spectral region 100–4400 cm^−1^, and Ge, covering the spectral region 600–4400 cm^−1^ as IRE, are shown in Fig. 4a. Qualitatively these spectra are globally similar to each other and to published ATR spectra of liquid water in the MIR region (e.g., MacDonald and Bureau^13^ and Elderderi et al.),^23^ but to the author’s knowledge, none of the previously reported ATR spectra of water covered the far-infrared spectral region. The direct observation of the two spectra in Fig. 4a reveals that they differ in the absolute intensity and also in the shape or position of some bands, as expected from the arguments presented in the Measurement Configuration and Spectrum Modeling section above. That is the case of the large wavenumber band around 3400 cm^−1^, which shows a slightly different profile in both spectra, and the band around 1640 cm^−1^, which has minimum values differing by about 6 cm^−1^ (Fig. 4a, insets).

The ATR spectra were modeled by using Eqs. 4–9 to account for the reflection response of the system, together with Eq. 11 to describe the dielectric function of water. The incidence angle taken in Eqs. 4–6 is the nominal value of 45°. Bearing in mind that the used beam is partially polarized, with an average fraction of about 83% of unpolarized light, the utilization of Eq. 9 (valid for completely unpolarized light) is an approximation.

The modeling was achieved by fitting the experimental data to Eq. 9 with an algorithm implemented within the free software GNU Octave.^24^ It should be noticed that Octave, like other modern programming languages, offer packages with algorithms that allow the manipulation of complex numbers and curve fitting with minimum effort, reducing the coding process very much. The fits were performed with the Levenberg–Marquardt algorithm for non-linear regression^25^ by using the Octave function “leasqr”.

The fitted curves (*R*_fit_) are depicted in Fig. 4a, together with the experimental spectra (*R*_exp_). To perform the fits, 14 modes (56 parameters) and 10 modes (40 parameters) of the factorized form of dielectric function (Eq. 11) are considered for spectra acquired with diamond and Ge ATR crystals, respectively. A large number of fitting parameters enables a faithful simulation of the experimental curves. The fitting parameters are shown in Tables A1 and A2 of the Appendix. Notice that the high-wavenumber dielectric constant, ε_∞_, cannot be an adjustable parameter in ATR. Whereas in external air/sample reflection ε_∞_ can be inferred from the highest wavenumber transparent region of the spectrum, in ATR this is not possible because the reflection is total in this spectral range. The employed value is ε_∞_ = 1.74, the square of the refractive index of water at about 9000 cm^−1^ taken from Querry et al.^26^ The purpose of the modeling is just to extract the dielectric function of the sample, with no intention of attributing any physical meaning to the fitting parameters. This procedure leads to an excellent fitting of the spectra, as can be seen from a direct comparison of experimental and calculated curves (Fig. 4a) and the plot of the residuals, *R*_exp_ – *R*_fit_, (Figs. 4b and 4c). Although the residuals are not randomly distributed all over the entire wavenumber range, showing a structure in some regions, their values are less than 1%.

A possible source of error in the previous analysis is the effect of beam spread due to the focusing of light into the IRE. In fact, the line representing the path of light in Fig. 1 is only valid for the central ray of the beam, whereas for the other rays in the beam a symmetric distribution of angles of incidence centered near 45° is expected. Because of the non-linear dependence of reflectance on the angle of incidence, the effective result can be described by an equivalent (effective) angle of incidence that is different from the nominal value.^14^ For the ATR accessory used in the present work, the details of the distribution of angles above mentioned, namely, its width, are not known. For another ATR system, Milosevic et al.^14^ estimated that an effective angle of 44.7° should be used rather than the nominal value of 45°. To roughly evaluate the effect of the beam spread in the results of the present work, a similar deviation is considered: simulated spectra for angles of incidence of 44.7° and 45° are compared, keeping the fitting parameters of Tables A1 and A2. It is found that in the case of Ge ATR crystal the relative deviation between the two spectra, (*R*(45°)–*R*(44.7°))/*R*(45°), is less than 0.4% for the whole spectral range (600–4400 cm^−1^), whereas for the case of diamond ATR crystal the relative deviations are clearly larger, attaining a maximum value of 4.5% at the lowest wavenumber, but with values less than 1% in most of the entire spectral range (100–4400 cm^−1^). The graphs of the relative deviations for both situations are depicted in Figure S1 (Supplemental Material).

Another possible cause of error in the modeling procedure is related to the assumptions of no dispersion of the refractive index and complete transparency of the IRE. The literature data indicate that going from 500 to 4000 cm^−1^ the refractive index of diamond increases about 0.006.^27^ So, only for the purpose of estimating a maximum error, it is reasonable to assume that from 100 cm^−1^ to 4400 cm^−1^ the variation of the refractive index is at most twice, that is, from 2.394 to 2.406. This variation can affect essentially the wavenumber extremities of the spectrum. The simulated reflectance at low wavenumber using *n* = 2.394 is smaller than that obtained by taking *n* = 2.40 and the relative deviation is smaller than 3%. On the other end of the spectrum, at high wavenumber, the simulated reflectance using *n* = 2.406 is higher than that obtained by taking *n* = 2.40 and is more important at the minimum of the band around 3400 cm^−1^, but nevertheless, the relative deviation is smaller than 1%. For Ge ATR crystal, the situation is similar. The refractive index of germanium varies from *n* = 4.00086 at 600 cm^−1^ to *n* = 4.092 at 4500 cm^−1^.^28^ The range that may be affected by this variation with respect to the taken value of *n* = 4.00 is the highest wavenumber region. The most pronounced effect of introducing *n* = 4.092 in the simulation is an increase of the reflectance at the minimum of the band around 3400 cm^−1^, with a relative deviation smaller than about 1%.

Diamond and Ge present very small absorptions in the studied spectral ranges, which have minor effects on the spectra. This was confirmed by using a complex refractive index for the IRE in the simulation of the spectra, considering the maximum possible values of the imaginary refractive index (*k* = 5.21 × 10^−4^, that is attained at 2016 cm^−1^ for diamond,^27^ and *k* = 1.71 × 10^−4^, that is attained at 645 cm^−1^ for Ge).^28^ The changes with respect to the simulations performed by considering a real refractive index are negligible (the relative deviations are less than 0.1% for diamond and less than 0.003% for Ge).

**Figure 5.**
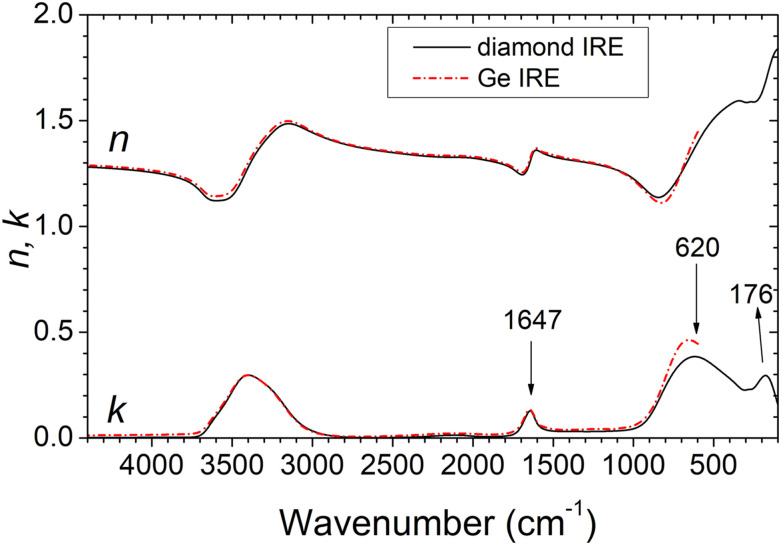
Real (*n*) and imaginary (*k*) refractive index of water retrieved from the spectra acquired with germanium and diamond internal reflection elements. For the experimental data, fitted spectra, and optical functions of water, see the research data files in the Supplemental Material.

Figure 5 gives the retrieved real and imaginary parts of refractive index of water. The results from the two measurements agree in the whole spectral range, except between 600 cm^−1^ and 900 cm^−1^. Still, globally there is an excellent quantitative reproducibility of results when the ATR crystal is changed.

The location of the main peaks of the imaginary refractive index (*k*) allows us to identify the principal infrared modes of water reported in the literature.^29,30^ The large, strong, irregularly shaped, absorption band around 3400 cm^−1^ is due to ν_1_ and ν_3_ modes corresponding to OH stretching vibrations, the ν_2_ mode associated with HOH bending mode is assigned to the absorption band at 1647 cm^−1^, the band at 620 cm^−1^ is ascribed to the libration of water molecules and the band at 176 cm^−1^ can be attributed to a translational mode.

Notice that the absorption frequencies could not be performed rigorously by direct inspection of the ATR spectra. This is just an example of the importance of modeling the ATR spectra and extracting the real and imaginary parts of the refractive index. Additionally, a careful analysis of the intensities, widths, and shapes of the optical functions all can provide useful information. Alternatively, depending on the particular material to be studied, the fitting can be performed with a model that incorporates itself parameters with precise physical meaning.

**Figure 6.**
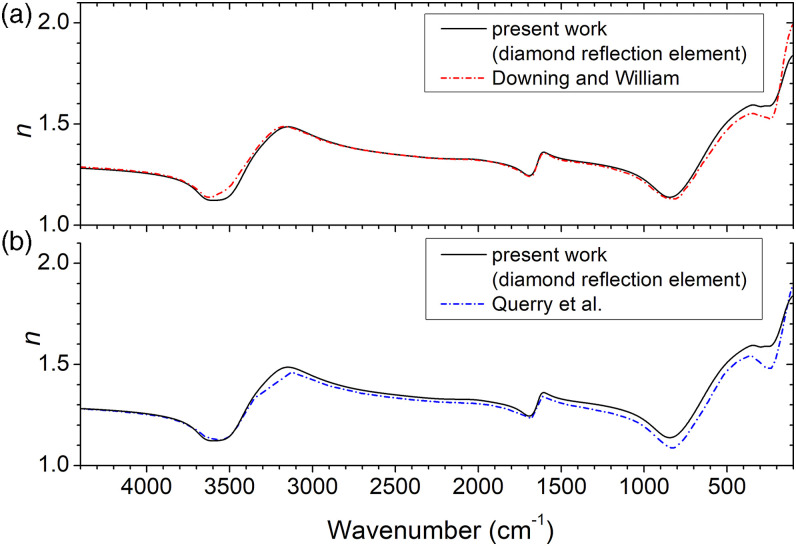
Index of refraction (*n*) obtained in the present work from modeling the spectrum acquired with diamond crystal as internal reflection element compared with results from (a) Downing and Williams^19^ and (b) Querry et al.^26^ For the experimental data, fitted spectra, and optical functions of water, see the research data files in the Supplemental Material.

Figure 6 shows the real part of the refractive index in comparison with those of Querry et al.^26^ and Downing and Williams,^19**.**^ who have compiled data from several sources. The two sets of literature data illustrate the magnitude of the differences that might be encountered among distinct measurements. There is an excellent global agreement between data of the present work and the reference results, especially with respect to Downing and Williams.^19^ However, at the low-wavenumber end (below 500 cm^−1^) there is a discrepancy with both reference curves, though qualitatively in accordance. Nevertheless, the agreement in the remaining regions, above 500 cm^−1^, is quite satisfactory, demonstrating the high reliability of the method to determine the optical functions from the ATR spectrum. In practical terms, this means that the determination of the optical functions of materials can be much simplified with the use of ATR conjugated with the described fitting method (because ATR removes some complexities of data acquisition in comparison with the more traditional techniques). The example of water illustrates well this point. The optical functions of water determined by Downing and Williams,^19^ based on transmission and standard specular reflectance at near normal incidence, required much more experimental effort than the ATR measurements of the present study. Yet, the method here presented is limited by the quality of contact between the sample and the IRE and requires the knowledge of the refractive index in the high-wavenumber region where the sample is transparent.

## Conclusion

Recognizing the reflection characteristics of the ATR technique is fundamental to a competent interpretation of ATR spectra. These features are illustrated by calculating the non-polarized reflectivity at the interface with the sample medium for the ATR configuration for various refractive indices of the IRE.

Following the method of spectral fitting based on Fresnel equations and dielectric dispersion models, it is possible to model the ATR spectra in a completely general way. With this approach, the spectroscopist can analyze any spectrum acquired in ATR configuration to extract the optical functions without worrying if specific conditions like the incidence angle, the relative refractive index of the sample with respect to the IRE, or the intensity of absorption bands, are satisfied. The method needs the input of the refractive index at high wavenumber (usually at optical frequencies), which must be known from an independent measurement. Of course, the accuracy of the results is limited by the quality of the recorded spectra. The fitting of ATR measurements in liquid water (obtained with diamond and Ge as IRE) here used to illustrate and test the method, provides a refractive index function that is in excellent quantitative agreement with values reported in the literature for the middle infrared region (500–4400 cm^−1^), but only qualitative accordance is observed for the lowest wavenumber infrared region (100 and 500 cm^−1^).

At present, the easy access to computing tools with powerful numerical capabilities and low effort coding (in which the programming language used in this work, that is, Octave, is just one example) give the means to retrieve accurate dielectric functions by examining the ATR spectra in an extended spectral range through the use of the standard method to fit reflectance spectra.

## Supplemental Material

Supplemental Material - Infrared Optical Functions of Water Retrieved Using Attenuated Total Reflection Spectroscopy Click here for additional data file. Supplemental Material for Infrared Optical Functions of Water Retrieved Using Attenuated Total Reflection Spectroscopy by Luis G. Vieira in Applied Spectroscopy

## Appendix

Tables A1 and A2 show the fitting parameters used to model the ATR spectra of water with the factorized form of the dielectric function. The parameters are identified with the symbols used in Eq. 11, but no physical meaning should be attributed to them.

**Table A1.** Parameters of Eq. 11 used for fitting the attenuated total reflection spectrum of water measured with diamond IRE. The high-wavenumber dielectric constant was taken as ε_∞_ = 1.74.

Ω_*P*_ (cm^−1^)	γ_*P*_ (cm^−1^)	Ω_*Z*_ (cm^−1^)	γ_*Z*_ (cm^−1^)
163.76	178.82	195.76	223.77
279.71	84.34	281.65	89.39
313.86	144.93	304.81	145.63
662.83	556.22	785.53	594.65
891.50	341.70	868.47	267.80
1381.22	328.95	1382.28	329.45
1633.67	73.35	1637.42	77.22
1679.83	114.19	1682.26	105.93
2008.56	252.55	2008.09	255.28
2226.19	510.88	2227.37	503.90
2910.94	485.55	2909.36	488.93
3203.24	341.52	3223.76	396.75
3443.49	302.72	3485.57	261.54
3655.46	163.96	3653.06	150.24

**Table A2.** Parameters of Eq. 11 used for fitting the attenuated total reflection spectrum of water measured with Ge IRE. The high-wavenumber dielectric constant was taken as ε_∞_ = 1.74.

Ω_*P*_ (cm^−1^)	γ_*P*_ (cm^−1^)	Ω_*Z*_ (cm^−1^)	γ_*Z*_ (cm^−1^)
741.91	431.47	1141.57	677.74
1111.78	630.19	813.64	277.78
1115.74	95.82	1115.45	95.26
1064.88	6988.42	1389.28	7226.38
1595.63	54.60	1594.91	54.68
1650.51	103.12	1659.60	99.06
2107.83	536.77	2114.82	541.94
3199.76	372.22	3219.44	447.38
3452.98	312.67	3491.52	268.22
3653.28	159.97	3651.86	147.87

## References

[1] Harrick N.J.. Internal Reflection Spectroscopy. New York: Wiley, 1967.

[2] Milosevic M.. Internal Reflection and ATR Spectroscopy. Hoboken, New Jersey: Wiley, 2012.

[3] Mirabella F.M. Jr. “History of Internal Reflection Spectroscopy”. In: Mirabella F.M., editor. Internal Reflection Spectroscopy: Theory and Applications. New York: Dekker, 1993. Chap. 1, Pp. 1‐15.

[4] Fahrenport J.. “Attenuated Total Reflection: A New Principle for the Production of Useful Infra-Red Reflection Spectra of Organic Compounds”. Spectrochim. Acta. 1961. 17(7): 698. 10.1016/0371-1951(61)80136-7

[5] Hancer M. Sperline R.P. Miller J.D.. “Anomalous Dispersion Effects in the IR-ATR Spectroscopy of Water”. Appl. Spectrosc. 2000. 54(1): 138-143. 10.1366/0003702001948222

[6] Belali R. Vigoureux J.-M. Morvan J.. “Dispersion Effects on Infrared Spectra in Attenuated Total Reflection”. J. Opt. Soc. Am. B. 1995. 12(12): 2377-2381. 10.1364/JOSAB.12.002377

[7] Spitzer W.G. Kleinman D.A.. “Infrared Lattice Bands of Quartz”. Phys. Rev. 1961. 121(5): 1324-1335. 10.1103/PhysRev.121.1324

[8] Spitzer W.G. Kleinman D. Walsh D.. “Infrared Properties of Hexagonal Silicon Carbide”. Phys. Rev. 1959. 113(1): 127-132. 10.1103/PhysRev.113.127

[9] Barker A.S.. “Infrared Lattice Vibrations and Dielectric Dispersion in Corundum”. Phys. Rev. 1963, 132(4): 1474-1481. 10.1103/PhysRevB.16.1717

[10] Berreman D.W. Unterwald F.C.. “Adjusting Poles and Zeros of Dielectric Dispersion to Fit Reststrahlen of PrCl_3_ and LaCl_3”_. Phys. Rev. 1968. 174(3): 791-799. 10.1103/PhysRev.174.791

[11] Gervais F. Piriou B.. “Anharmonicity in Several-Polar-Mode Crystals: Adjusting Phonon Self-Energy of LO and TO Modes in Al_2_O_3_ and TiO_2_ to Fit Infrared Reflectivity”. J. Phys. C: Solid State Phys. 1974. 7(13): 2374-2386. 10.1088/0022-3719/7/13/017

[12] Meneses D.S. Brun J.F. Echegut P. Simon P.. “Contribution of Semi-Quantum Dielectric Function Models to the Analysis of Infrared Spectra”. Appl. Spectrosc. 2004. 58(8): 969-974. 10.1366/0003702041655467 18070390

[13] MacDonald S.A. Bureau B.. “Fourier Transform Infrared Attenuated Total Reflection and Transmission Spectra Studied by Dispersion Analysis”. Appl. Spectrosc. 2003. 57(3): 282-287. 10.1366/000370203321558182 14658619

[14] Milosevic M. Wendland N. Lee R.E. Gregory B.W.. “The Usefulness of Spectroscopic Simulations”. Appl. Spectrosc. 2020. 74(3): 305-313. 10.1177/0003702819893689 31746219

[15] Piro O.E. Castellano E.E. Gonzalez S.R.. “Attenuated Total-Reflectance Spectra of Strongly Absorbing Anisotropic Single Crystals: Trigonal α-Quartz”. Phys. Rev. B: Solid State. 1988. 38(12), 8437-8443. 10.1103/PHYSREVB.38.8437 9945603

[16] Guida J.A. Piro O.E. Castellano E.E. Aymonino P.J.. “Attenuated Total Reflectance Infrared Spectra of Strongly Absorbing Anisotropic Crystals: Orthorhombic Na_2_[Fe(CN)_5_NO]_2_H_2_O”. J. Chem. Phys. 1989. 91(7): 4265-4272. 10.1063/1.456806

[17] Balan E. Mauri F. Lemaire C. Brouder C., et al. “Multiple Ionic Plasmon Resonances in Naturally Occurring Multiwall Nanotubes: Infrared Spectra of Chrysotile Asbestos”. Phys. Rev. Lett. 2002. 89(17): 177401. 10.1103/PhysRevLett.89.177401 12398703

[18] Aufort J. Ségalen L. Gervais C. Brouder C., et al. “Modeling the Attenuated Total Reflectance Infrared (ATR-FTIR) Spectrum of Apatite”. Phys. Chem. Miner. 2016. 43: 615-626. 10.1007/s00269-016-0821-x

[19] Downing H.D. Williams D. Querry M.R.. “Optical Constants of Water in the Infrared”. J. Geophys. Res. 1975. 80(12): 1656-1661. 10.1364/JOSA.61.000895

[20] Born M. Wolf E.. Principles of Optics: Electromagnetic Theory of Propagation, Interference, and Diffraction of Light. Cambridge, UK: Cambridge University Press, 1980.

[21] Lucarini V. Saarinen J.J. Peiponen K.-E. Vartiainen E.M.. Kramers–Kronig Relations in Optical Materials Research. Berlin: Springer, 2005.

[22] Chipman R.A. Lam W.-S.T. Young G.. Polarized Light and Optical Systems. New York: CRC Press, 2019. Pp. 66‐67.

[23] Elderderi S. Leman-Loubiére C. Wills L. Henry S., et al. “ATR-IR Spectroscopy for Rapid Quantification of Water Content in Deep Eutectic Solvents”. J. Mol. Liq. 2020. 311: 113361. 10.1016/j.molliq.2020.113361

[24] Eaton J.W. Bateman D. Hauberg S. Wehbring R.. GNU Octave, v.6.1.0 Manual. https://docs.octave.org/v6.1.0/ [accessed Sep 8 2022].

[25] Press W.H. Teukolsky S.A. Vetterling W.T. Flannery B.P.. Numerical Recipes: The Art of Scientific Computing. Cambridge, UK: Cambridge University Press, 2007. Pp. 801.

[26] Querry M.R. Wieliczka D.M. Segelstein D.J.. "Subpart 2: Semiconductors. Water (H_2_O)". In: Palik E.D., editor. Handbook of Optical Constants of Solids. San Diego: Academic Press, 1998. Vol. 2, Pp. 1059–1077.

[27] Edwards D.F. Philipp H.R.. "Subpart 3: Insulators. Cubic Carbon (Diamond)". In: Palik E.D., editor. Handbook of Optical Constants of Solids. San Diego: Academic Press, 1998. Vol. 1, Pp. 665‐674.

[28] Potter R.F.. "Subpart 2: Semiconductors. Germanium (Ge)". In: Palik E.D., editor. Handbook of Optical Constants of Solids. San Diego, USA: Academic Press, 1998. Vol. 1, Pp. 465‐478.

[29] Williams D.. “Frequency Assignments in Infra-Red Spectrum of Water”. Nature. 1966. 210: 194-195. 10.1038/210194a0

[30] Eisenberg D. Kauzmann W.. The Structure and Properties of Water. Oxford; New York: Clarendon Press; Oxford University Press, 2005. Pp. 229.

