# Supplementary material for: Infrared Optical Functions of Water Retrieved Using Attenuated Total Reflection Spectroscopy
Source: Appl Spectrosc. 2022 Oct 5;77(2):178–86. doi: 10.1177/00037028221128813 (PMC9903249; doi:10.1177/00037028221128813)
Supplement: Supplemental Material - Infrared Optical Functions of Water Retrieved Using Attenuated Total Reflection Spectroscopy [file sj-pdf-1-asp-10.1177_00037028221128813.pdf]

Supplemental Material

Infrared Optical Functions Retrieved from Water Using Attenuated Total Reflection

Luis G. Vieira[a]*

[a]Centro de Física das Universidades do Minho e do Porto (CF-UM-UP), Laboratório de Física para Materiais e Tecnologias Emergentes (LaPMET) and Departamento de Física, Universidade do Minho, Campus de Gualtar, Braga 4710-057, Portugal

*Corresponding author email: lvieira@fisica.uminho.pt

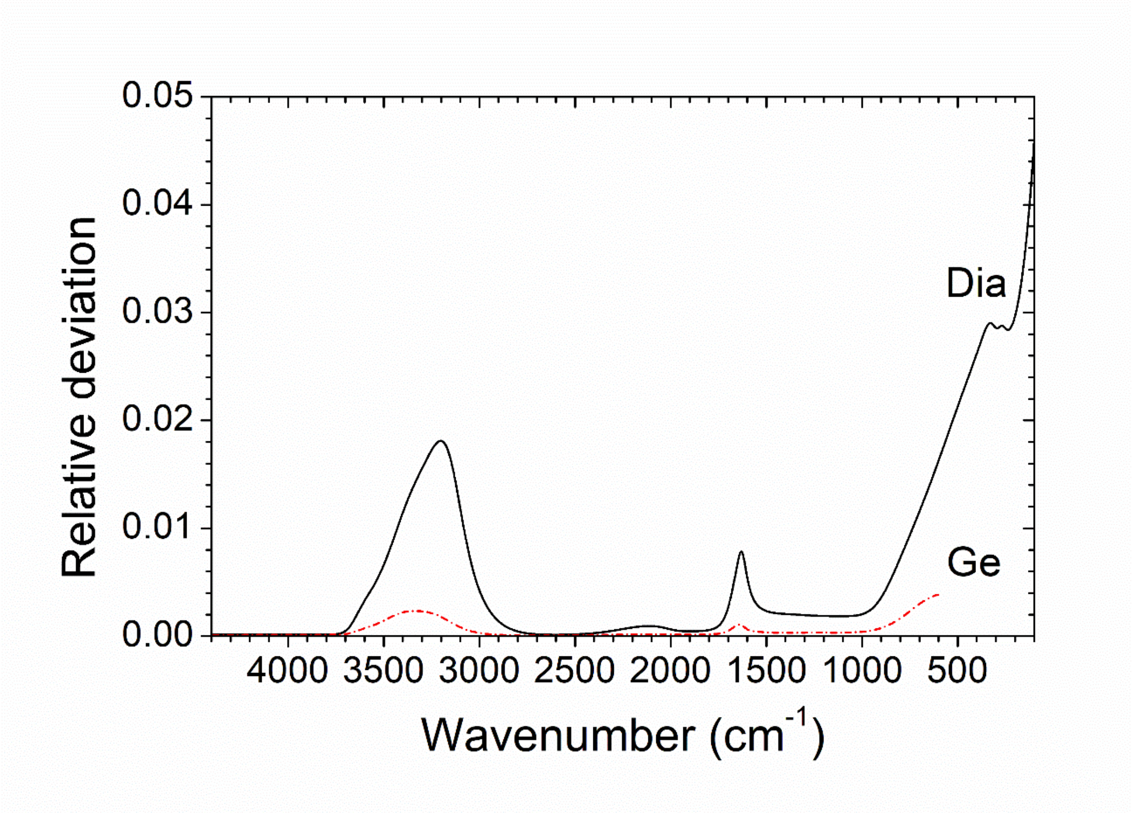

Figure S1. Effect of beam spread on the ATR spectra of water obtained with diamond (Dia) and germanium (Ge) as internal reflection elements. The relative deviation is calculated from the relative difference between spectra simulated for a nominal angle of incidence of 45° and an effective angle of incidence of 44.7°, i.e., $(R(45^{\circ}) - R(44.7^{\circ}))/R(45^{\circ})$.
